# Low Survival Rates of Oral and Oropharyngeal Squamous Cell Carcinoma

**DOI:** 10.1155/2017/5815493

**Published:** 2017-05-30

**Authors:** Anna Carolina Omena Vasconcellos Le Campion, Camila Maria Beder Ribeiro, Ronir Raggio Luiz, Francisco Feliciano da Silva Júnior, Herbert Charles Silva Barros, Karine de Cássia Batista dos Santos, Stefania Jeronimo Ferreira, Lucio Souza Gonçalves, Sonia Maria Soares Ferreira

**Affiliations:** ^1^CESMAC University Center, Maceió, AL, Brazil; ^2^Federal University of Alagoas, Maceió, AL, Brazil; ^3^Federal University of Rio de Janeiro, Rio de Janeiro, RJ, Brazil; ^4^State Secretary of Health (SESAU), Alagoas, Brazil; ^5^Post-Graduate Program in Dentistry, Estácio de Sá University, Rio de Janeiro, RJ, Brazil

## Abstract

**Aim:**

To assess the epidemiological and clinical factors that influence the prognosis of oral and oropharyngeal squamous cell carcinoma (SCC).

**Methods:**

One hundred and twenty-one cases of oral and oropharyngeal SCC were selected. The survival curves for each variable were estimated using the Kaplan-Meier method. The Cox regression model was applied to assess the effect of the variables on survival.

**Results:**

Cancers at an advanced stage were observed in 103 patients (85.1%). Cancers on the tongue were more frequent (23.1%). The survival analysis was 59.9% in one year, 40.7% in two years, and 27.8% in 5 years. There was a significant low survival rate linked to alcohol intake (*p* = 0.038), advanced cancer staging (*p* = 0.003), and procedures without surgery (*p* < 0.001). When these variables were included in the Cox regression model only surgery procedures (*p* = 0.005) demonstrated a significant effect on survival.

**Conclusion:**

The findings suggest that patients who underwent surgery had a greater survival rate compared with those that did not. The low survival rates and the high percentage of patients diagnosed at advanced stages demonstrate that oral and oropharyngeal cancer patients should receive more attention.

## 1. Introduction

The incidence of oral cancer in Brazil has been estimated at around 15,290 new cases per year, putting oral cancer into the seventh position of all malignant neoplasia [1]. Among several histological types of oral neoplasia, the squamous cell carcinoma (SCC) is the more frequent, representing 90% of all cases of oral cancer [2].

Cancer is primarily a disease caused by genetic changes that progress as sequential series of somatic mutations in specific genes such as protooncogenes and tumor suppressor genes, resulting in uncontrolled cancerous cell proliferation [3, 4]. These events can be triggered by extrinsic and/or intrinsic factors, such as lifestyle, environmental, immunosuppression, and individual susceptibility [5, 6]. Among the extrinsic factors, tobacco use and alcohol intake represent the highest risk to the emergence of this malignant disease [7, 8].

Studies have shown that low social and economic status and deprivation are significantly associated with an increased risk of oral cancer [9, 10].

The primary location of oral cancer is an important prognostic factor because the affected anatomic area can determine the accessibility and extension of surgery [11]. In addition, it can define the necessity of additional therapeutic procedures, such as the prophylactic cervical ganglionectomy as well as radiotherapy and adjuvant chemotherapy [12].

Almost half of the cases of oral cancer worldwide have been diagnosed in stages III and IV [13, 14]. Early diagnosis of oral and oropharyngeal SCC is essential for a good prognosis and therefore dentists play a fundamental role in the early detection and prevention of this type of oral cancer [15].

Oral and oropharyngeal cancer has a survival rate ≤50% after 5 years. However, this time can be increased when the cancers are diagnosed at an early stage [16, 17]. Cancer mortality is influenced by variations in quantity and quality of the available health services, the delay in diagnosis, or diagnoses at advanced stages, beyond the sequelae of treatment [18]. Oral cancer is a public health problem and early diagnosis as well as assurance of appropriated and fast care is required in order to achieve a better quality of life and lower morbidity/mortality for these patients. Thus, the aim of the current study was to assess the influence of epidemiological and clinical factors on the prognostic of oral and oropharyngeal squamous cell carcinoma (SCC) in a group of patients from one referral center in the state of Alagoas, Brazil.

## 2. Material and Methods

One hundred and twenty-one patients diagnosed in a reference service in stomatology as having oral and oropharyngeal squamous cell carcinoma (SCC) and treated in an Oncology High Complexity Center (CACON) (Alagoas, Brazil) were enrolled in the current study. As the inclusion criteria, all patients must be diagnosed and treated only in these two centers. All subjects were informed about the aims, risks, and benefits of the study and signed a consent form. Patients older than 18 years were examined between March 2005 and March 2013. The medical records whose lack of information made the results of the study unfeasible were excluded.

The study protocol was conducted in full accordance with the World Medical Association Declaration of Helsinki and was approved by the Review Committee for Human Subjects of the University Center of Research CESMAC (number 367.585/2013).

### 2.1. Procedures and Instruments

Epidemiological data (age, gender, skin color, residence, education, and occupation), risk habits (alcohol intake and tobacco use), clinical characteristics, treatment implemented for the oral cancer, date of the diagnosis, date of the last appointment, and date of death were collected from medical records and during the patient's appointments with the examiner.

The date of death was confirmed using the Mortality Information System applying the following filters: patient's name, date of birth, and mother's name. Date of death as well as the underlying cause was searched according to the International Statistical Classification of Diseases and Related Health Problems-10th Revision (ICD-10).

The oral cancers were located on the hard palate, gingiva, anterior two-thirds of tongue, lips, jugal mucosa, floor of mouth, and alveolar and retromolar mucosa, while the oropharyngeal cancers were on the soft palate, tongue base, tonsillar region, uvula, and posterior pharynx [2]. The clinical stage evaluation was carried out according to the Union for International Cancer Control [19].

### 2.2. Data Analysis

All analyses were performed using the software SPSS®20.0 for Windows (Statistical Package for Social Sciences, IBM, USA). The descriptive analysis included the absolute and relative frequency for categorical variables. Comparison between groups (death and survival) was carried out using the Chi-square test or Fisher's exact test.

For the inferential analysis with the outcome death, the following variables were changed: the anatomical locations of cancers were clustered as oral and oropharyngeal cancers; TNM system was categorized as early stage (I and II) and advanced stage (III and IV); treatment was categorized as no surgery [radiotherapy (RT), chemotherapy (CT), or combination RT/CT] and surgery (surgery alone, surgery + RT, and surgery + RT + CT).

In order to assess the survival, all the deaths during the study were considered outcome. The variables for the survival analysis were age, gender, skin color, occupation, residence, education, tobacco use, alcohol intake, cancer location, cancer staging, and surgery procedures. The survival curve was estimated for each variable using the Kaplan-Meier method. The comparison between curves was obtained by the long-rank test. The Cox regression model was used to assess the effect of the variables on survival (multivariate analysis to calculate hazard ratios), which included variables with the following characteristics according to the Kaplan-Meier analysis: significant difference (*p* < 0.05) and no crossing between curves. The level of significance established for all analyses was 5%.

## 3. Results

### 3.1. Characteristics of the Study Sample

The sociodemographic and clinical characteristics of the 121 patients who participated in the current study are presented in Table 1. The majority of the patients were male (*n* = 81; 66.9%), >40 years old (*n* = 77; 63.7%), and with white skin color (*n* = 47; 38.8%). The frequencies of tobacco use and alcohol intake were 111 (91.7%) and 77 (63.6%), respectively. Cancers at an advanced stage were observed in 103 patients (85.1%). Cancers on the tongue were the most frequent (23.1%), followed by mouth floor (18.2%) and hard and soft palate (16.5%). Of the patients who underwent surgery (*n* = 22; 18.2%), 59% were diagnosed in advanced stages (III and IV).

Of the 121 patients, 82 (67.8%) died due to SCC and only one (1.2%) was <40 years old. The majority (*n* = 57; 69.5%) were male, illiterate (*n* = 63; 76.8%), noneconomically active (*n* = 52; 63.4%), smokers (*n* = 78; 95.1%), alcohol users (*n* = 56; 68.3%), with advanced cancer staging (*n* = 74; 90.2%), and not undergoing any surgical procedures (*n* = 75; 91.5%). When the sociodemographic and clinical characteristics were compared between dead and alive patients, significant difference was found only in the cancer staging (*p* = 0.009) and surgery procedures (*p* < 0.001) (Table 2).

### 3.2. Survival Analysis

**Figure 1:**
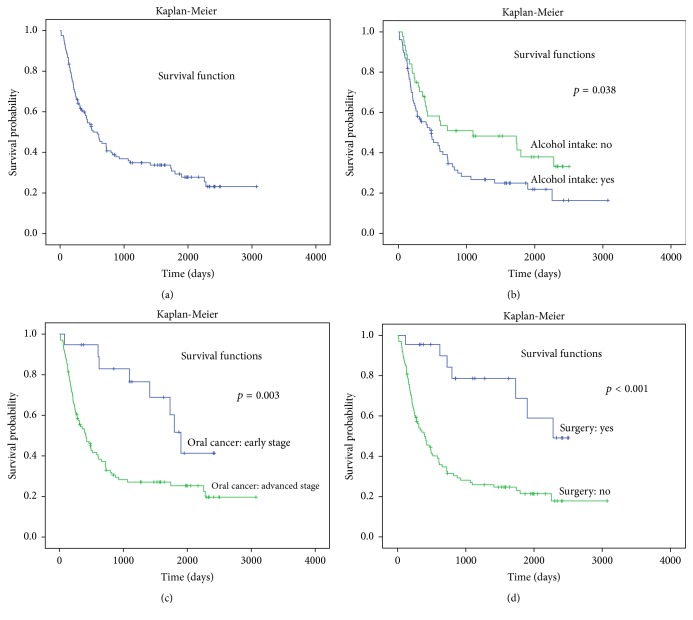
(a) Survival curve of patients with oral and oropharyngeal squamous cell carcinoma estimated by Kaplan-Meier method (Maceió, AL, Brazil, 2005–2013). (b) Survival curve for alcohol intake of patients with oral and oropharyngeal squamous cell carcinoma estimated by Kaplan-Meier method (Maceió, AL, Brazil, 2005–2013). (c) Survival curve for cancer staging of patients with oral and oropharyngeal squamous cell carcinoma estimated by Kaplan-Meier method (Maceió, AL, Brazil, 2005–2013). (d) Survival curve for surgical procedure of patients with oral and oropharyngeal squamous cell carcinoma estimated by Kaplan-Meier method (Maceió, AL, Brazil, 2005–2013).

The survival analysis using Kaplan-Meier method was 59.9% in one year, 40.7% in two years, and 27.8% in 5 years. The mortality rate of the sample was 3.1/100,000 per year. The mean survival was 1165.7 days (3.2 years) (95% CI: 940.5–1390.9). The median survival was 515 days (95% CI: 323.3–703.8); that is, half of the patients had died by 515 days after diagnosis (Figure 1(a)).

The long-rank test demonstrated significant low survival for alcohol intake (*p* = 0.038), advanced cancer staging (*p* = 0.003), and no surgical procedures (*p* < 0.001). The survival probability and respective curve regarding these variables are presented in Figures 1(b)–1(d). In the first year after diagnosis, the survival probabilities were 55.3% and 68.0% for alcohol users and non-alcohol users, respectively, while in the fifth year the probabilities were 21.8% and 37.9%. The median survival was 484 days (95% CI: 240.1–727.9) for alcohol users and 1095 (95% CI: 0–2646.8) for non-alcohol users (Figure 1(b)). In the fifth year, the survival probability was 51.6% and 25.3% for patients with early stage (I and II) and advanced stage (III and II) of cancer, respectively. The median survival was 1,900 days (95% CI: 1647.1–2152.9) for early stage of cancer and 415 days (95% CI: 251.9–578.1) for the advanced stage patients (Figure 1(c)). The death rate was 1.1/100,000 subjects per year for early stage and 3.8/100,000 subjects per year for advanced stage patients.

The survival probability of patients who underwent surgical procedures (associated with chemotherapy, radiotherapy, or both) was 84.2% (second year) and 59.0% (fifth year). On the other hand, the patients that did not receive any surgical procedures presented lower survival probabilities: 31.5% (second year) and 21.4% (fifth year) (Figure 1(d)). The median survival was 2,280 days for the patients who underwent surgery, while for patients that did not receive any surgical procedures the median survival was 395 days (95% CI: 326.3–703.8).

### 3.3. Multivariate Analysis

The variables alcohol intake, cancer staging, and surgery were included in the Cox regression model. Of the three variables, only surgical procedures (*p* = 0.005) demonstrated significant effect on the survival. In addition, the outcome of death was 3 times faster for the patients who did not receive any surgery compared to those that did (Table 3).

## 4. Discussion

Oral cancer is a public health problem and its frequency has increased in recent decades. SCC is the most frequently observed histological cancer type in the mouth and, in virtue of the regional metastasis, has demonstrated a high mortality rate [1, 20]. In the current study, clinical characteristics, types of treatment, and epidemiological findings were similar to other studies [21–24]. All deaths were caused by oral or oropharyngeal SCC with a frequency (67.8%) higher than that found by Arriagada et al. [25] in a Chilean population (41.6%).

In the present study, the percentage of patients diagnosed with oral SCC who survived 5 years (27.8%) was similar to Santos et al. [26] (28.4%) and De Oliveira et al. [27] (24%); however, it was lower than other studies, such as Bórquez et al. [23] (57%), Arriagada et al. [25] (58.4%), Honorato et al. [28] (43.1%), Montoro et al. [29] (39%), and De Araújo Daher et al. [30] (38.71%).

The findings of the current study show that patients in Alagoas with SCC died after a mean of 3.2 years (1165.7 days). The mean survival was higher than the results observed by Honorato et al. [28] (882 days). However, the median survival (515 days) was lower than that found by Miyamoto et al. [31] (930 days) and De Araújo Daher et al. [30] (690 days). Diagnoses in the Northeast region of Brazil are late which leads to cancers with advanced staging, which may favor this low survival rate.

The findings of the present study for cancer staging were similar to other studies. De Araújo Daher et al. [30] demonstrated that survival was better in stage I. After 5 years, the authors observed that the survival rate of the patients with stage II was 59.7%, stage III 41.6%, and stage IV 23.9%. Santos et al. [32] reported mean survival higher in patients with stage II (10 years), while in stage IV it was 3.7 years. In the study by Bórquez et al. [23], the survival for stages I, II, II, and IV was 86%, 67%, 52%, and 51%, respectively. These findings confirm the early diagnosis of oral cancer as an important step in increasing survival rates. In addition, the early treatment of a primary neoplasm reduces the mortality, notably before its dissemination. Oral SCC has a high rate of cure (around 80%) up to the fifth year with stage I of the disease (T1N0), whereas with stage IV it is only 20% [16, 33]. Therefore, the prevention of oral cancer, earlier diagnosis, and active treatment of early stage disease may be the best means of improving 5-year survival rates and quality of life after treatment. Achieving these goals may require the enforcement of public education and social efforts relevant to early diagnosis through regular oral examinations by expert clinicians [34].

Surgical treatment is the preferred method for radical treatment, and neck dissection can be performed simultaneously when there is a suspicion of neck lymph node metastasis. In cases of early stage disease where there is no neck metastasis, a single-modality surgical treatment or radiotherapy is appropriate [35].

The present study demonstrated that patients submitted to surgical treatment had a higher survival rate. In the study of Honorato et al. [28] there were a number of similarities with the findings observed here, such as the relevance of surgical treatment, as well as the absence of affected lymph nodes at the moment of diagnosis as a condition necessary for longer survival. In the study of Shan and Gil [35], complete resection of the lesion had the best results in early stage oral cancer. In advanced oral cancer, there was no significant difference between chemotherapy and/or radiotherapy and surgery in terms of 5-year survival rates. However, functional and aesthetic defects that result from radical surgery can reduce a patient's quality of life. Therefore, conservative methods are preferred over radical treatment in older patients.

There is no significant difference between the cancer location and survival in the current study and similar results were observed by De Oliveira et al. [27] However, Arriagada et al. [25] demonstrated a higher survival rate for patients who had cancer in the lower inferior lip when compared with other oral regions. Moreover, for De Araújo Daher et al. [30] the percentage of death was higher in cancers diagnosed in the oropharynx (73.7%) than in an oral location (50.8%), suggesting the possibility that SCC that take place in the posterior anatomic location can go unnoticed for a longer time, delaying the diagnosis. Honorato et al. [28] showed that cancers located in the hard palate and jugal mucosa presented the worst prognosis. Thus, there is a disagreement with respect to the localization of the oral neoplasm and the prognosis.

In the present study, there was no association between survival and the variables gender, ethnicity, and tobacco use, after Kaplan-Meier analysis. These findings are similar to those observed by Honorato et al. [28] On the other hand other authors have found associations between survival and different variables. For instance, Miyamoto et al. [31] performed a multivariate analysis and observed an association of advanced cancer (HR 2.5) and RT and CT (HR 4.1) with survival. Montoro et al. [29] reported that the cervical metastasis from oral SCC and inadequate surgical margins affected the survival of patients. In a retrospective study, Matos et al. [36] assessed 57 patients diagnosed with oral SCC (except in lips) and found that tumoral thickness >10 mm represents an independent risk factor for early progression of oral SCC after surgical treatment.

The epidemiological findings with respect to the deaths were similar to those found by Santos et al. [32]. These authors evaluated the profile of the patients who died between 2000 and 2009 in Aracaju (Capital of Sergipe state, Brazil) and observed that majority of the deaths (seventy-eight) were males, 50–60 years old, with brown skin color, with low education, and from neighborhoods with low quality of life. In the current study, the comparison between survival and nonsurvival groups demonstrated significant difference for cancer staging (*p* = 0.009) and surgical procedures (*p* < 0.001). The findings showed a high likelihood of death in patients with advanced cancer staging and without surgical treatment. In the multivariate model, the risk factor was confirmed only for the absence of surgical procedures (*p* = 0.005). However, Honorato et al. [28] observed association of death with other variables, such as ethnicity (*p* = 0.033), cancer located in the lower gingiva (*p* = 0.048), and treatment (*p* < 0.0001). On the present study surgical treatment increased survival even in the advanced stages. Better 5-year survival rate with patients who underwent surgery was reported also in other studies [28, 37] evidencing greater survival in those patients undergoing this procedure.

## 5. Conclusion

The low survival rates and the large percentage of patients with an advanced cancer staging diagnosis, as well as the findings that showed an improved survival of those patients who underwent surgery, reinforce the need for greater attention to be paid to oral and oropharyngeal cancer, especially among high-risk populations (elderly, smokers, and alcohol users). In order to increase the 5-year survival rate of oral and oropharyngeal carcinoma, it may be necessary to improve public education and social efforts relevant to early diagnosis.

## Figures and Tables

**Table 1:** Sociodemographic and clinical characteristics of the 121 patients.

Variable	*N*	%
Age		
<40 years	44	36.3
40 to 70 years	37	30.6
>70 years	40	33.1
Gender		
Male	81	66.9
Female	40	33.1
Skin color		
White	47	38.8
Brown	41	33.9
Black	33	27.3
Residence		
Urban	72	59.5
Rural	49	40.5
Education		
Illiterate	87	71.9
Literate	34	28.1
Occupation		
Economically active	48	39.7
Noneconomically active	51	42.1
Not informed	22	18.2
Tobacco use		
Yes	111	91.7
No	10	8.3
Alcohol intake		
Yes	77	63.6
No	44	36.4
Cancer location		
Tongue (anterior two-thirds)	28	23.1
Mouth floor	22	18.2
Hard palate	20	16.5
Alveolar ridge	12	9.9
Oropharynx	12	9.9
Other	27	22.4
Clinical presentation		
Ulcer	76	62.8
Nodule	17	14.0
Ulcerative and verrucose	10	8.3
Ulcerated nodules	06	5.0
Not informed	12	9.9
Cancer staging		
Early	18	14.9
Advanced	103	85.1
Surgery		
Yes	22	18.2
No	99	81.8

**Table 2:** Comparison of studied variables between patients with and without death (oral cancer/SCC) after diagnosis of oral and oropharyngeal cancer.

Variable	Death		*p* value
	Yes (*N* = 82)	No (*N* = 39)	
*N* (*%*)			
Age^¶^			0.434
<40 years	1 (1.2)	2 (5.1)	
40 to 70 years	55 (67.1)	25 (64.1)	
>70 years	26 (31.7)	12 (30.8)	
Gender^*∗*^			0.384
Female	25 (30.5)	15 (38.5)	
Male	57 (69.5)	24 (61.5)	
Skin color^*∗*^			0.270
White	23 (28.0)	10 (25.6)	
Brown	35 (42.7)	12 (30.8)	
Black	24 (29.3)	17 (43.6)	
Residence^*∗*^			0.659
Urban	35 (42.7)	15 (38.5)	
Rural	47 (57.3)	24 (61.5)	
Education^*∗*^			0.236
Illiterate	63 (76.8)	23 (66.7)	
Literate	19 (23.2)	13 (33.3)	
Occupation^*∗*^			0,665
EA	30 (36.6)	18 (46.2)	
NEA	52 (63.4)	21 (53.8)	
Tobacco use^¶^			**0.050**
Yes	78 (95.1)	33 (84.6)	
No	04 (4.9)	6 (15.4)	
Alcohol intake			0.123
Yes	56 (68.3)	21 (53.8)	
No	26 (31.7)	18 (46.2)	
Cancer location^¶^			0.334
Oral	72 (87.8)	37 (94.9)	
Oropharynx	10 (12.2)	02 (5.1)	
Cancer staging^¶^			**0.009**
Early	08 (9.8)	11 (28.2)	
Advanced	74 (90.2)	28 (71.8)	
Surgery^¶^			**<0.001**
Yes	07 (8.5)	15 (38.5)	
No	75 (91.5)	24 (61.5)	

^*∗*^Chi-square test. ^¶^Fisher's exact test. NEA: noneconomically active; EA: economically active.

**Table 3:** Cox regression analysis between three variables studied and survival.

Variable	*β*	Standard error	HR	95% CI	*p* value
Alcohol intake	0.29	0.25	1.34	0.83–2.16	0.240
Cancer staging	0.60	0.40	1.82	0.84–3.95	0.130
Surgical procedure	1.15	0.41	3.17	1.41–7.12	**0.005**

HR = hazard ratio.

## References

[1] Instituto Nacional do Câncer (INCA) (2014). *Incidência de Câncer no Brasil, Estimativa 2014*.

[2] Neville B., Damm D. D., Allen C. M., St. Louis (2009). *Oral and Maxillofacial Pathology*.

[3] Wang Z., Wang C., Zhao Z. (2013). Association between −251A>T polymorphism in the interleukin-8 gene and oral cancer risk: a meta-analysis. *Gene*.

[4] Li W., Chen J., Liu C. (2013). Glutathione S-transferase P1 Ile105Val polymorphism and oral cancer risk: a meta-analysis. *International Journal of Medical Sciences*.

[5] Petti S. (2009). Lifestyle risk factors for oral cancer. *Oral Oncology*.

[6] Warnakulasuriya S. (2009). Causes of oral cancer—an appraisal of controversies. *British Dental Journal*.

[7] Hindle I., Downer M. C., Moles D. R., Speight P. M. (2000). Is alcohol responsible for more intra-oral cancer?. *Oral Oncology*.

[8] Ferreira Antunes J. L., Toporcov T. N., Biazevic M. G. H., Boing A. F., Scully C., Petti S. (2013). Joint and independent effects of alcohol drinking and tobacco smoking on oral cancer: a large case-control study. *PLoS ONE*.

[9] Conway D. I., Petticrew M., Marlborough H., Berthiller J., Hashibe M., Macpherson L. M. D. (2008). Socioeconomic inequalities and oral cancer risk: a systematic review and meta-analysis of case-control studies. *International Journal of Cancer*.

[10] Ferreira M. A. F., Gomes M. N., Michels F. A. S., Dantas A. A., Latorre M. D. R. D. D. O. (2012). Social inequality in morbidity and mortality from oral and oropharyngeal cancer in the city of São Paulo, Brazil: 1997–2008. *Cadernos de Saude Publica*.

[11] Almeida F. C. S. A., Cazal C., Nunes F. D., Araújo M. E., Dias R. B., Silva D. P. (2011). Prognostic factors in oral cancer. *Rev Bras Cienc Saude*.

[12] Fan K.-H., Lin C.-Y., Kang C.-J. (2007). Combined-modality treatment for advanced oral tongue squamous cell carcinoma. *International Journal of Radiation Oncology Biology Physics*.

[13] Warnakulasuriya S. (2009). Global epidemiology of oral and oropharyngeal cancer. *Oral Oncology*.

[14] Güneri P., Epstein J. B. (2014). Late stage diagnosis of oral cancer: components and possible solutions. *Oral Oncology*.

[15] Akbulut N., Oztas B., Kursun S., Evirgen S. (2011). Delayed diagnosis of oral squamous cell carcinoma: a case series. *Journal of Medical Case Reports*.

[16] Rogers S. N., Brown J. S., Woolgar J. A. (2009). Survival following primary surgery for oral cancer. *Oral Oncology*.

[17] Kowalski L. P., Carvalho A. L., Martins Priante A. V., Magrin J. (2005). Predictive factors for distant metastasis from oral and oropharyngeal squamous cell carcinoma. *Oral Oncology*.

[18] Biazevic M. G. H., Castellanos R. A., Antunes J. L. F., Michel-Crosato E. (2006). Trends in oral cancer mortality in the city of São Paulo, Brazil, 1980–2002. *Cad Saude Publica*.

[19] Sobin L. H., Gospodarowicz M. K., Wittekind C. *Union for International Cancer Control—UICC*.

[20] Warnakulasuriya S. (2010). Living with oral cancer: epidemiology with particular reference to prevalence and life-style changes that influence survival. *Oral Oncology*.

[21] dos Santos L. C. O., de Medeiros Batista O., Cangussu M. C. T. (2010). Characterization of oral cancer diagnostic delay in the state of Alagoas. *Brazilian Journal of Otorhinolaryngology*.

[22] Aui A. L. R. O., Tanimoto H. M., Queiroz C. D. S. (2012). Oral and oropharynges neoplasm—a transversal study in Pio XII Foundation—Cancer Hospital of Barretos. *Rev Odontol UNESP*.

[23] Bórquez P., Capdeville F., Madrid A., Veloso M., Cßrcamo M. (2011). Analysis of survival of 137 patients with oral cancer. *Rev Chil Cir*.

[24] Ferraz L. G., Fávero E., Franzi A. S., Rapoport A., Curioni A. O. (2010). Relationship between the clinical (TNM) and histopathological (pTNM) staging with survival in advanced squamous cell carcinoma of the mouth and oropharynx. *Revista Brasileira de Cirurgia de Cabeça e Pescoço*.

[25] Arriagada O. C., Venegas B. R., Cantín M. L., Zavando D. M., Manterola C. D., Suazo I. G. (2010). Oral squamous cell carcinoma: retrospective analysis of 36 cases.. *Revista chilena de cirugía*.

[26] Santos M. A., Danesi C. C., Pinheiro B. H. (2014). Relationship between survival of patients with squamous cell carcinoma of the oral cavity and pathological staging, operated at the University Hospital of the Federal University of Santa RS.Maria. *Revista Brasileira de Cirurgia de Cabeça e Pescoço*.

[27] De Oliveira L. R., Ribeiro-Silva A., Zucoloto S. (2006). Incidence and survival profile of patients with oral squamous cell carcinoma in a Brazilian population. *Jornal Brasileiro de Patologia e Medicina Laboratorial*.

[28] Honorato J., Camisasca D. R., Silva L. E., Dias F. L., Faria P. A. S., Lourenço S. Q. C. (2009). Overall survival analysis in oral squamous cell carcinoma patients diagnosed at the National Cancer Institute. *Revista Brasileira de Epidemiologia*.

[29] Montoro J. R. D. M. C., Hicz H. A., De Souza L. (2008). Prognostic factors in squamous cell carcinoma of the oral cavity. *Brazilian Journal of Otorhinolaryngology*.

[30] De Araújo Daher G. C., De Araújo Pereira G., Oliveira A. C. D. (2008). Epidemiological characteristics of cases of mouth cancer registered in a hospital in the city of uberaba from 1999–2003: a warning toward the need for early diagnosis. *Revista Brasileira de Epidemiologia*.

[31] Miyamoto K. N., Bruhn R. F., Rosa D. S., Capelli F. A., Kanda J. L. (2014). Treatment of oropharyngeal squamous cell cancer with chemotherapy and radiotherapy.Rev Bras Cir Cabeça e Pescoço. *Kanda JL. Treatment of oropharyngeal squamous cell cancer with chemotherapy and radiotherapy.Rev Bras Cir Cabeça e Pescoço*.

[32] Santos V. T. G., Santos V. S., Cravalho R. A. S., Guedes S. A. G., Trento C. L. (2013). Mortality from oral cancer in Aracaju/SE. *Aracaju/SE*.

[33] van der Waal I., de Bree R., Brakenhoff R., Coebergh J.-W. (2011). Early diagnosis in primary oral cancer: is it possible?. *Medicina Oral, Patologia Oral y Cirugia Bucal*.

[34] Seo B., Lee C., Kim J. (2016). Changes in the management and survival rates of patients with oral cancer: a 30-year single-institution study. *Journal of Korean Association of Oral Maxillofacial Surgeons*.

[35] Shan J. P., Gil Z. (2009). Current concepts in management of oral cancer: surgery. *Oral Oncology*.

[36] Matos L. L., Pinto F. R., Kulcsar M. A. V. (2014). Tumor thickness as an independent risk factor of early recurrence in oral cavity squamous cell carcinoma. *Rev Bras Cir Cabeça e Pescoço*.

[37] Geum D., Roh Y., Yoon S. (2013). he impact factors on 5-year survival rate in patients operated with oral cancer. *J Korean Assoc Oral Maxillofac Surg*.

